# The association between caesarean section and childhood asthma: an updated systematic review and meta-analysis

**DOI:** 10.1186/s13223-019-0367-9

**Published:** 2019-10-29

**Authors:** Behzad Darabi, Shoboo Rahmati, Mohammad Reza HafeziAhmadi, Gholamreza Badfar, Milad Azami

**Affiliations:** 1 Department of Pediatrics, Faculty of Medicine, Ilam University of Medical Sciences, Ilam, Iran; 2 School of Public Heath, Ilam University of Medical Sciences, Ilam, Iran; 3 Faculty of Medicine, Ilam University of Medical Sciences, Ilam, Iran; 4 Department of Pediatrics, Behbahan Faculty of Medical Sciences, Behbahan, Iran; 5 School of Medicine, Ilam University of Medical Sciences, Ilam, Iran

**Keywords:** Asthma, Caesarean section, Meta-analysis

## Abstract

**Background:**

Investigating the association between caesarean section (SC) and childhood asthma has shown contradictory results in different studies. The present study was conducted to determine the association between SC and childhood asthma.

**Material and method:**

The present study was conducted based on the preferred reporting items for systematic reviews and meta-analyses (PRISMA) guidelines. All the steps of the study were conducted independently by two reviewers from the inception until February 1, 2019. In case of disagreement, the third reviewer resolved it. We searched international online databases, including Scopus, Cochrane Library, PubMed/Medline, Embase, Web of Science (ISI), Science Direct, and Google scholar. The results of studies were combined using random effects model, and heterogeneity was measured through I^2^ index and Cochran’s Q test. Comprehensive Meta-Analysis Software was used for meta-analysis. The significance level of all tests was considered to be P < 0.05.

**Results:**

The heterogeneity rate was high (I^2^ = 67.31%, P < 0.001) in 37 studies. The results showed that SC increased the risk of childhood asthma (RR (relative risk) = 1.20 [95% CI 1.15–1.25, P < 0.001]). The association between emergency and elective SC and childhood asthma was significant with RR of 1.18 (95% CI 1.07–1.29, P < 0.001) in 13 studies and 1.23 (95% CI 1.20–1.26, P < 0.001) in 13 studies, respectively. The subgroup analysis for RR of childhood asthma in SC indicated that study design (P = 0.306), prospective/retrospective studies (P = 0.470), quality of studies (P = 0.514), continent (P = 0.757), age of diagnosis (P = 0.283) and year of publication (P = 0.185) were not effective in the heterogeneity of studies. Sensitivity analysis by removing one study at a time indicated that the overall estimate is robust.

**Conclusion:**

According to the meta-analysis, SC (overall, elective, and emergency) increased the risk of childhood asthma. Therefore, it is hoped that developing clinical guidelines and implementing appropriate management plans would diminish the risk of asthma.

## Introduction

Asthma is one of the most common airway diseases, which involves increased response of tracheobronchial tree to various stimuli. Asthma attacks may last from a couple of minutes to a couple of hours. Continuous asthma includes successive obstructed airways, which lasts for several days or weeks [1]. The incidence of asthma is higher among people under 18 years of age and the hospitalization of children suffering from asthma is continuously rising [2]. The prevalence, incidence, mortality, and economic burden of asthma have increased since 1960, especially among children [3]. The incidence of asthma has increased in developed countries, South Africa, Eastern Europe, and Baltic countries, though this increase mainly occured in two populations (children and the elderly) [3–5]. The highest prevalence of asthma is in UK, New Zealand, Australia, Ireland, Canada (all above 14%), and the United States (11%); the prevalence of asthma has doubled in Western Europe over the last two decades. Asthma is the most common cause of children’s hospitalization in Europe [3]. Asthma is multifactorial, and this implies that asthma is the result of poverty and other environmental factors, smoking, air pollution, congestion, dust, house pets, psychological factors, lack of access to hygiene, genetics, history of viral infection, and low birth weight [6, 7]. Type of delivery is another contributing factor that causes allergic diseases, such as asthma. Therefore, the present study investigates the association between caesarean section (CS) and childhood asthma [7–12]. Various mechanisms have been proposed regarding the impact of delivery mode on asthma; mechanisms such as mechanical effects on lung growth, immunological mechanisms, and their impact on intestinal flora [12]. During vaginal delivery, the infant gets in touch with probiotics and the microbiome that may affect the development of atopic diseases [13]. There have been many studies all around the world on the association between CS and childhood asthma, and a meta-analysis was conducted in 2006 that reported significant association between CS and childhood asthma by analyzing 23 studies [14]. Through reviewing and synthesizing all related documents, systematic review and meta-analysis can present a more comprehensive picture of a problem in the community [15, 16]. Therefore, another study is required to represent a more panoramic image of this issue all over the world; thus, the present systematic study was conducted to investigate the association between CS and children asthma.

## Methods

### Study protocol

The present study was conducted based on the preferred reporting items for systematic reviews and meta-analyses (PRISMA) [17]. All steps of the study were conducted independently by two researchers (M.A and Sh.R). In case of disagreement, the third researcher resolved it.

### Search strategy

We searched international online databases, including Scopus, Cochrane Library, PubMed/Medline, Embase, Web of Science (ISI), Science Direct, and Google scholar from the inception until February 1, 2019. Search was preformed based on the following keywords: “Cesarean Section”[MeSH], “Asthma”[MeSH], “Child”[MeSH], and “Mode of Delivery” [Text word]. References of the searched articles were reviewed to ensure literature saturation on the topic.

### Definitions

The definition of asthma was based on physician’s diagnosis, hospitalization for asthma, medication use for asthma, asthma reported by the child/patient, his or her parents, or both, and the history of asthma. Elective CS is a planned CS designed for pregnant women for any maternal or embryonic indications before the onset of labor. The emergency CS in the women’s emergency care department is generally defined after the onset of labor.

### Qualitative assessment

The modified Newcastle–Ottawa Scale (NOS) for non-randomized studies was used to conduct qualitative assessment [18]. This checklist covered 4 criteria, which includes 8 sections. Finally, the two researchers compared the points given to each article. The minimum acceptable score was considered 5. The total score of NOS was 10 and the qualified articles were divided into three groups of low quality (0 to 4), medium quality (5 to 7), and high quality (8 to 10).

### Inclusion and exclusion criteria

Inclusion criteria [19] were determined with respect to prospective and retrospective studies that assessed SC and childhood asthma.

Exclusion criteria of the present meta-analysis were: (1) studies that did not focus on the SC as the exposure and childhood asthma as outcome; (2) duplicated studies; (3) non-English full text; (4) non-accessible full text [i.e. articles that were not available through my Institution]; (5) review articles, case reports, letters to the editor, comments, or conference papers; and (6) low quality studies according to NOS checklist.

### Selection of studies

At the end of the search, the articles were entered into the EndNote software, and after the “Find References Updates”, duplicate studies were omitted. After blinding the studies (hiding the name of the authors, the name of the journal and the year of publication), each study was evaluated independently by two authors at the screening stage (by reading titles and abstracts), through scanning the titles of studies and evaluating the inclusion and exclusion criteria (the eligibility stage). In case of disagreement between the two researchers, the expert researcher made the final decision.

### Data extraction

First, a checklist was designed according to the objectives of the study. The designed checklist included the following: the name of the author(s), the year of publication, the location of the study, the sample size, the duration of the follow-up, relative risk (RR), odds ratio (OR) with 95% confidence interval (CI), age category, and the number of events in both groups (case and control), which was extracted by two independent researchers, while the name of the author, the institute and the journal was blinded. If necessary, further details and the raw data were requested by contacting the author (the first author, the corresponding author or the group of authors).

### Statistical analysis

Cochran’s Q test and I^2^ index were used to evaluate the heterogeneity of the studies. There are four categories for I^2^ index: I^2^ index below 25% is low heterogeneity, 25–50% is moderate heterogeneity, 51–75% is substantial heterogeneity and above 75% is considerable heterogeneity. The random effects model was used to generate the pooled RR or OR and 95% CI in case of significant heterogeneity, otherwise, a fixed-effects model was used [20]. Subgroup analysis was used to find the cause of heterogeneity between the studies. Sensitivity analysis was used to measure the predictive power by excluding one study. Finally, the publication bias was investigated using funnel plot and Egger and Begg’s tests. Statistical analysis was performed using Comprehensive Meta-Analysis Software version 2. The significance level of the tests was considered to be P < 0.05.

## Results

### Search results

**Fig. 1:**
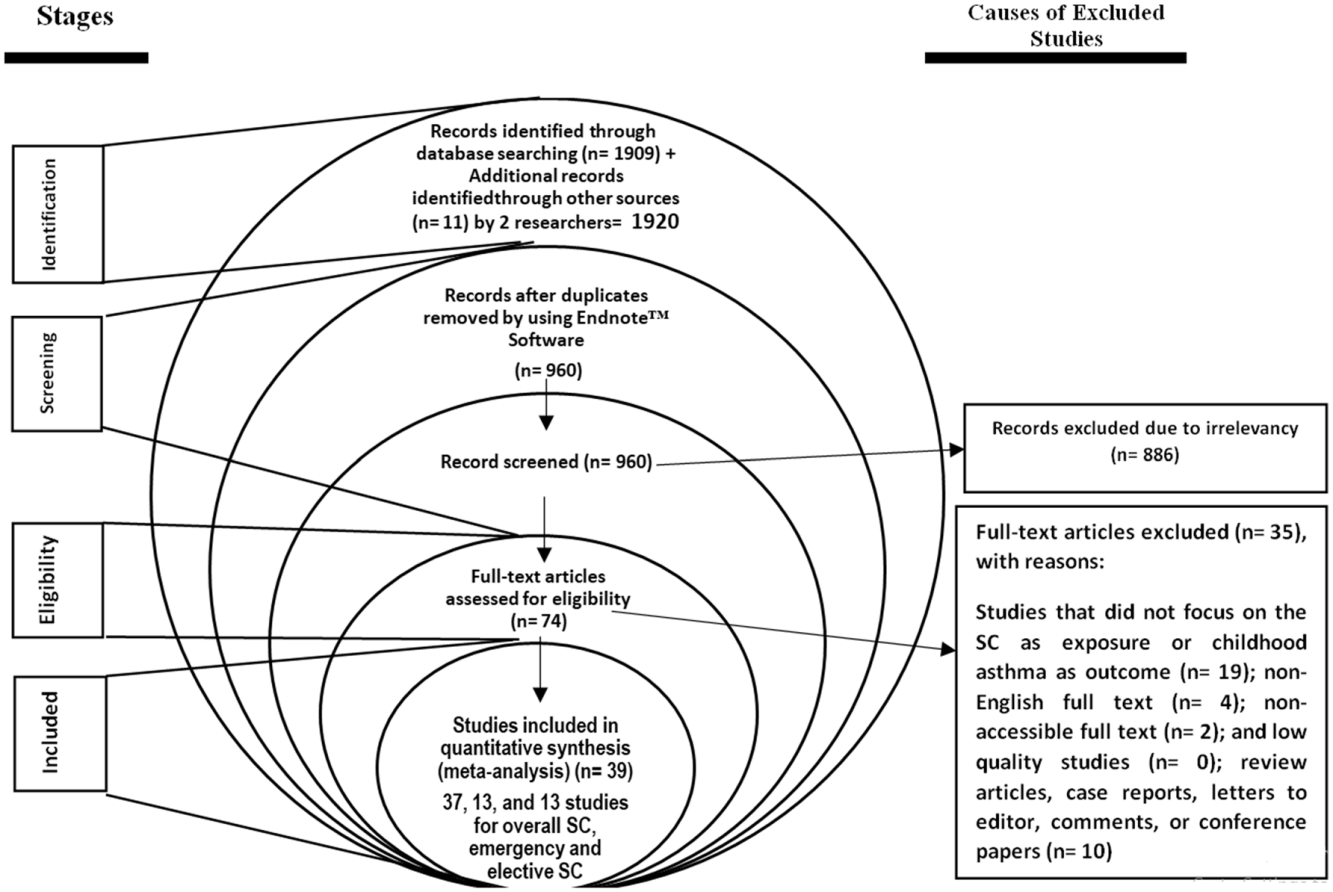
Meta-analysis flowchart

A total of 1909 studies were identified by two researchers by searching the databases and manual search identified eleven more studies, and 960 studies were excluded due to duplication. After screening the titles and abstracts, 886 studies were excluded due to irrelevancy and after assessing the full text, 35 studies were excluded due to lack of focus on the association between SC and childhood asthma (n = 19), non-English or non-accessible full text (n = 6), letters to the editor without original data, review article and case report (n = 10), and low quality (n = 0). Finally, 39 studies (37, 13, and 13 studies for overall SC, emergency and elective SC) with appropriate quality entered the meta-analysis (Fig. 1 and Table 1).

**Table 1:** Characteristics of studies qualified for meta-analysis

Authors, publication date, (reference)	Design	Country/continent	Age (years)	Asthma definition	Sample size	Year of birth	RR^a^ (95% CI^b^)	Quality of studies
Xu et al. 2000 [21]	Cohort prospective	Finland/Europe	7	Parental questionnaire (diagnosis)	8088	1985–1986	1.38 (1.00–1.92)	High
Nafstad et al. 2000 [22]	Cohort prospective	Norway/Europe	4	Questionnaire (diagnosis and symptoms)	2531	1992–1993	1.10 (0.70–1.80)	High
Kero et al. 2002 [8]	Cohort retrospective	Finland/Europe	7	57 years: hospital admissions and medications databases (ICD code); at 7 years: clinical visit (diagnosis)	59,927	1987–1995	1.21 (1.08–1.36)	High
McKeever et al. 2002 [11]	Cohort retrospective	UK/Europe	0–11	Parental questionnaire (diagnosis)	29,238	1993–1997	1.09 (1.01–1.18)	High
Bager et al. 2003 [9]	Cohort prospective	Denmark/Europe	< 28	Interview (diagnosis	9722	1973–1977	1.33 (1.02–1.74)	High
Hakansson et al. 2003 [10]	Cohort retrospective	Sweden/Europe	> 1	Hospital discharge records (ICD code)	316,918	1984–1996	1. 14 (1.07–1.22)	High
Maitra et al. 2004 [12]	Cohort prospective	UK/Europe	5–8	Parental questionnaire (diagnosis)	12,367	1991–1992	1.16 (0.90–1.50)	High
Smith et al. 2004 [23]	Cohort retrospective	Scotland/Europe	8–9	Hospital admissions (ICD code)	241,846	1992–1995	1.10 (1.00–1.20)	High
Renz-polster et al. 2005 [24]	Cohort prospective	US/USA	3–10	Medical records (diagnosis)	8953	1990–1992	1.24 (1.01–1.57)	High
Bernsen et al. 2005 [25]	Cohort retrospective	Netherlands/Europe	> 6	Medical records (diagnosis)	1961	1988–1990	1.03 (0.51–2.08)	High
Juhn et al. 2005 [26]	Cohort retrospective	UK/Europe	7	Medical records (diagnosis or symptoms)	7106	1976–1982	0.93 (0.60–1.40)	High
Delbey et al. 2005 [27]	Case–control retrospective	Washington/USA	6–12	Hospital admissions (ICD code)	10,320	–	1.20 (1.04–1.39)	High
Salam et al. 2006 [28]	Cohort retrospective	US/USA	< 18	Parental questionnaire (diagnosis)	6259	1975–1987	1.33 (1.01–1.75)	High
Werner et al. 2007 [29]	Cohort prospective	Danish/Europe	15–18	Parental questionnaire (diagnosis)	7119	1984–1987	1.11 (0.88–1.39)	High
Roduit et al. 2008 [30]	Cohort prospective	Netherlands/Europe	8	Parental questionnaire (diagnosis)	2917	1996–1997	1.79 (1.27–2.51)	High
Pistiner et al. 2008 [31]	Cohort prospective	US/USA	9	Parental questionnaire (diagnosis)	432	1994–1996	1.10 (0.60–2.30)	Moderate
Tollnes et al. 2008 [32]	Cohort retrospective	Norway/Europe	18	Parental questionnaire (diagnosis)	1,869,380	1967–1998	1.52 (1.42–1.62)	High
Metsala et al. 2008 [33]	Cohort retrospective	Finland/Europe	> 3	Hospital admissions (ICD code)	22,548	1996–2004	1.15 (1.05–1.25)	High
Mohammadzadeh et al. 2009 [34]	Case–control retrospective	Iran/Asia	3–14	Hospital admissions (ICD code)	512	–	1.20 (0.80–1.70)	Moderate
Devidson et al. 2010 [35]	Cohort retrospective	UK/Europe	2–11	Hospital admissions (ICD code)	248,612	1970–1989	1.18 (1.02–1.34)	High
Park et al. 2010 [36]	Cohort retrospective	Korea/Asia	≤ 16	Questionnaire (diagnosis and Symptoms)	279	2003	0.76 (0.37–1.57)	Moderate
Nimwegen et al. 2011 [37]	Cohort prospective	Netherlands/Europe	6–7	Parental questionnaire (diagnosis)	2343	2002	0.89 (0.50–1.56)	High
Magnus et al. 2011 [38]	Cohort prospective	Norway/Europe	3	Parental questionnaire (diagnosis)	37,171	1999–2008	1.15 (1.02–1.29)	High
Nathan et al. 2011 [39]	Case–control retrospective	Malaysian/Asia	3–15	Hospital admissions (ICD code)	156	–	1.17 (0.47–2.91)	Moderate
Almqvist et al. 2012 [40]	Cohort retrospective	Sweden/Europe	> 10	National patient register (ICD code)	87,500	1993–1999	1.20 (1.05–1.37)	High
Hancox et al. 2012 [41]	Cohort retrospective	New Zealand/Oceania	13	Hospital admissions (ICD code)	1037	1972–1973	0.92 (0.32–2.65)	High
Kolokotroni et al. 2012 [42]	Cross-sectional	Cyprus/Europe	8	Parental questionnaire (diagnosis)	2216	–	1.41 (1.09–1.83)	High
Braback et al. 2013 [43]**	Cohort retrospective	Sweden/Europe	2–5	Swedish prescribed Drug Register (Antiasthmatic drugs)	199,837	1999–2006	1.20 (1.13–1.28)	High
Braback et al. 2013 [43]**	Cohort retrospective	Sweden/Europe	6–9	Swedish prescribed Drug Register (Antiasthmatic drugs)	199,837	1999–2006	1.18 (1.09–1.27)	High
Guibas et al. 2013 [44]	Cross-sectional	Aetoloakarnania/Europe	9–13	Hospital admissions (ICD code)	2572	–	1.39 (1.04–1.87)	High
Van Berkel et al. 2015 [51]	Cohort prospective	Netherlands/Europe	6	Questionnaire (diagnosis)	6128	–	1.09 (0.76–1.55)	High
Black et al. 2016 [45]	Cohort retrospective	UK, Scotland/Europe	5	Hospital admissions (ICD code)	40,145	1993–2007	1.11 (0.99–1.25)^*^	High
Kristensen et al. 2016 [52]	Cohort	Denmark/Europe	0–14	Hospital admissions (ICD code)	750,569	1997–2012	1.12 (1.09–1.50)	High
Sevelsted et al. 2016 [53]	Cohort prospective	Denmark/Europe	0–15	–	864,049	1997–2010	2.18 (1.27–3.73)	High
Lavin et al. 2017 [46]**	Cohort prospective	India/Asia	8	Questionnaire (diagnosis)	2026	2001–2002	2.60 (1.30–5.40)	High
Lavin et al. 2017 [46]**	Cohort prospective	Vietnam/Asia	8	Questionnaire (diagnosis)	2000	2001–2002	2 (1.20–3.30)	High
Chu et al. 2017 [54]	Case–control retrospective	China/Asia	4–12	Questionnaire (diagnosis)	1385	2015–2016	–	High
Brix et al. 2017 [47]	Cohort retrospective	Denmark/Europe	0–15	(ICD code)	928	1997–2012	–	High
Rusconi et al. 2017 [55]	Cohort retrospective	European/Europe	5–9	Questionnaire (diagnosis	67,613	1996–2006	1.22 (1.02–1.46)	High

^a^Relative risk for overall cesarean section

^b^Confidence interval

* RR was calculated based on event and total numbers in case and control groups

** Some studies have been included and estimated the RR for more than one population or regions

### Association between caesarean section and childhood asthma

**Fig. 2:**
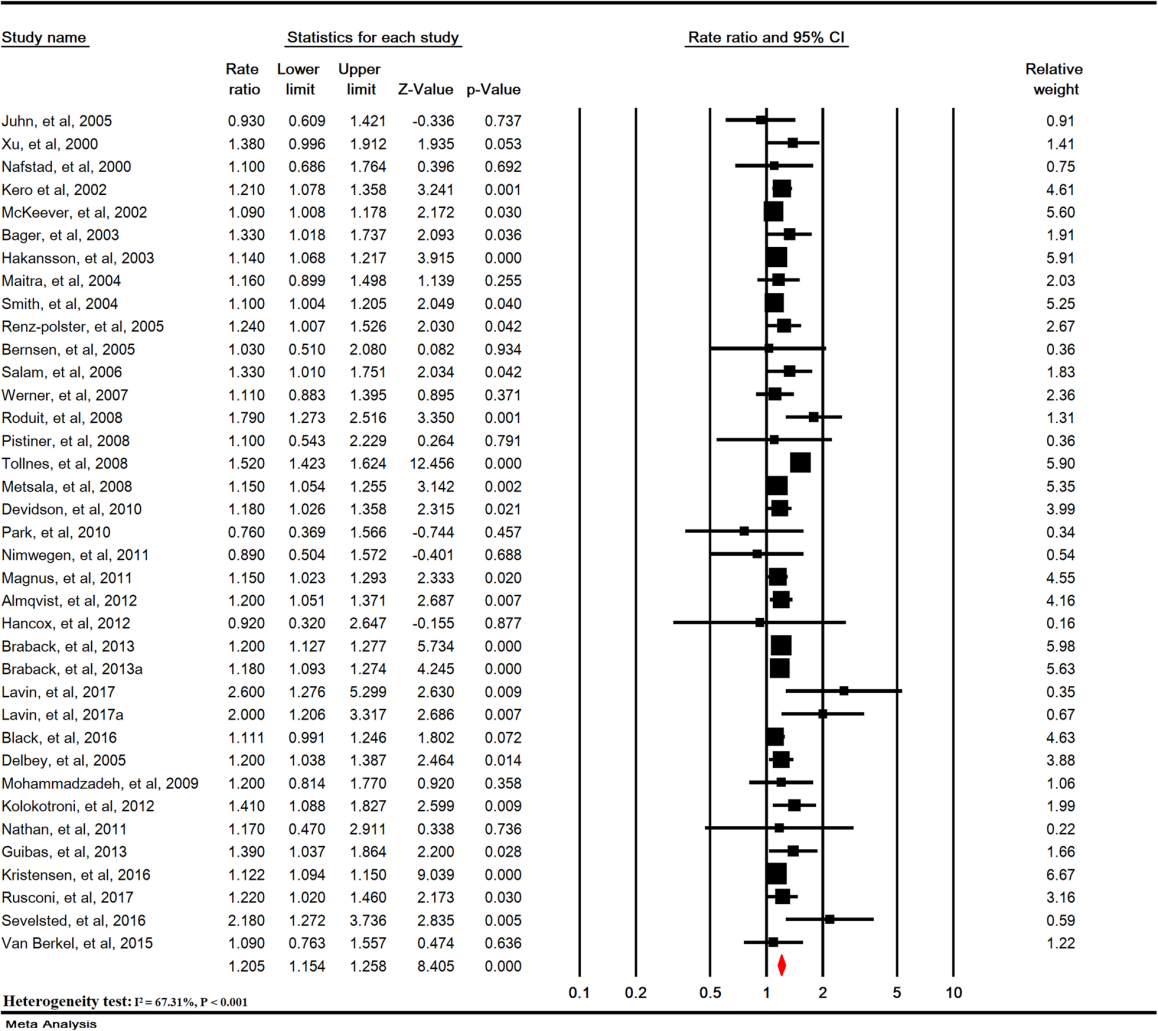
Relative risk of childhood asthma in cesarean section. Random-effects model

**Fig. 3:**
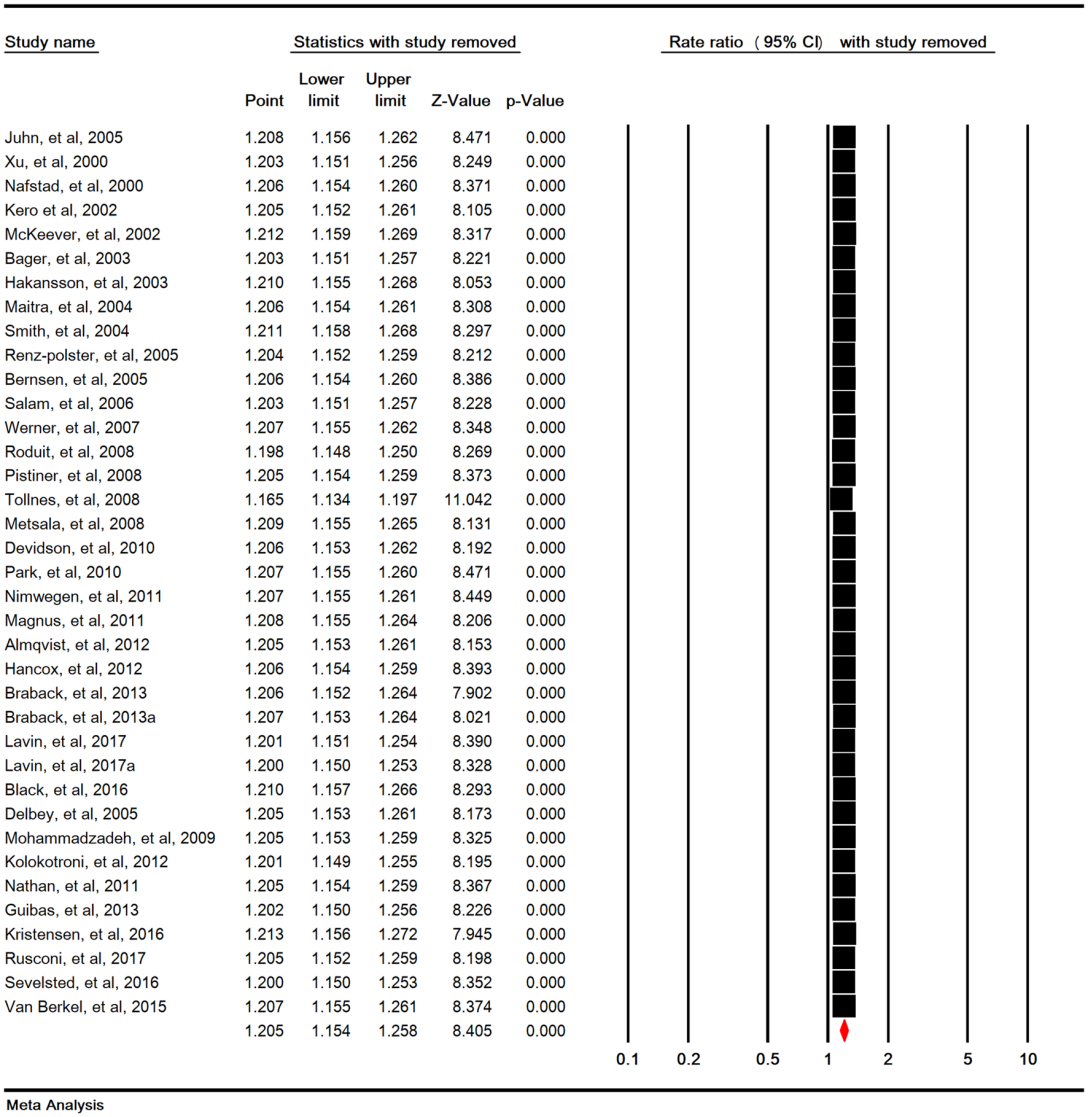
Sensitivity analysis for relative risks of childhood asthma in cesarean section

Heterogeneity rate was high (I^2^ = 67.31%, P < 0.001) in 37 studies. The association between SC and childhood asthma was significant (RR = 1.20 [95% CI 1.15–1.25, P < 0.001]) (Fig. 2). Sensitivity analysis by removing one study at a time indicated that the overall estimate is robust (Fig. 3).

### The subgroup analysis for caesarean section and childhood asthma

The subgroup analysis for RR of childhood asthma in SC indicated that study design (P = 0.306), prospective/retrospective studies (P = 0.470), quality of studies (P = 0.514), continent (P = 0.757), age of diagnosis (P = 0.283) and year of publication (P = 0.185) were not effective in the heterogeneity of studies (Table 2).

**Table 2:** The subgroup analysis for relative risks of childhood asthma in cesarean section

Variable		Studies (N^a^)	Sample (N)	Heterogeneity		RR^b^	95% CI^c^	*P* value
				I^2^	P-value			
Year of publication (year)	2000–2004	8	680,637	0	0.603	1.134	1.090–1.180	< 0.001
	2005–2009	11	1,937,507	73.652	< 0.001	1.256	1.115–1.415	< 0.001
	2010–2014	11	581,723	0	0.823	1.191	1.145–1.239	< 0.001
	2015–2018	7	1,866,897	64.631	0.009	1.234	1.089–1.397	< 0.001
	Test for subgroup differences: Q = 5.189, df(Q) = 3, P = 0.158							
Study design	Cohort	32	5,049,603	70.899	< 0.001	1.198	1.144–1.255	< 0.001
	Case–control	3	12,373	0	0.999	1.199	1.049–1.372	0.008
	Cross-sectional	2	4788	0	0.943	1.401	1.154–1.702	0.001
	Test for subgroup differences: Q = 2.367, df(Q) = 2, P = 0.306							
Prospective/retrospective	Prospective	15	1,716,415	49.38	0.016	1.242	1.135–1.360	< 0.001
	Retrospective	22	3,350,349	70.176	< 0.001	1.195	1.132–1.261	< 0.001
	Test for subgroup differences: Q = 0.523, df(Q) = 1, P = 0.470							
Quality of studies	High	33	4,931,946	70.57	< 0.001	1.167	1.148–1.187	< 0.001
	Moderate	4	134,818	0	0.749	1.095	0.818–1.465	0.544
	Test for subgroup differences: Q = 0.427, df(Q) = 1, P = 0.514							
Continent	USA	4	25,964	0	0.914	1.227	1.102–1.367	< 0.001
	Europe	27	4,901,351	73.451	< 0.001	1.197	1.142–1.254	< 0.001
	Asia	5	138,412	51.819	0.081	1.431	0.967–2.118	0.073
	Oceania	1	1037	0	NA	0.92	0.320–2.647	0.877
	Test for subgroup differences: Q = 1.182, df(Q) = 3, P = 0.757							
Age of diagnosis (years)	≤ 10	20	726,194	22.466	0.177	1.182	1.135–1.231	< 0.001
	> 10	4	2,097,090	80.450	0.002	1.274	1.045–1.555	0.017
	Both	13	2,243,480	14.038	0.303	1.141	1.101–1.182	< 0.001
	Test for subgroup differences: Q = 2.523, df(Q) = 2, P = 0.283							

^a^Number

^b^Relative risk

^c^Confidence interval

### Relative risks of childhood asthma in emergency and elective cesarean section

**Fig. 4:**
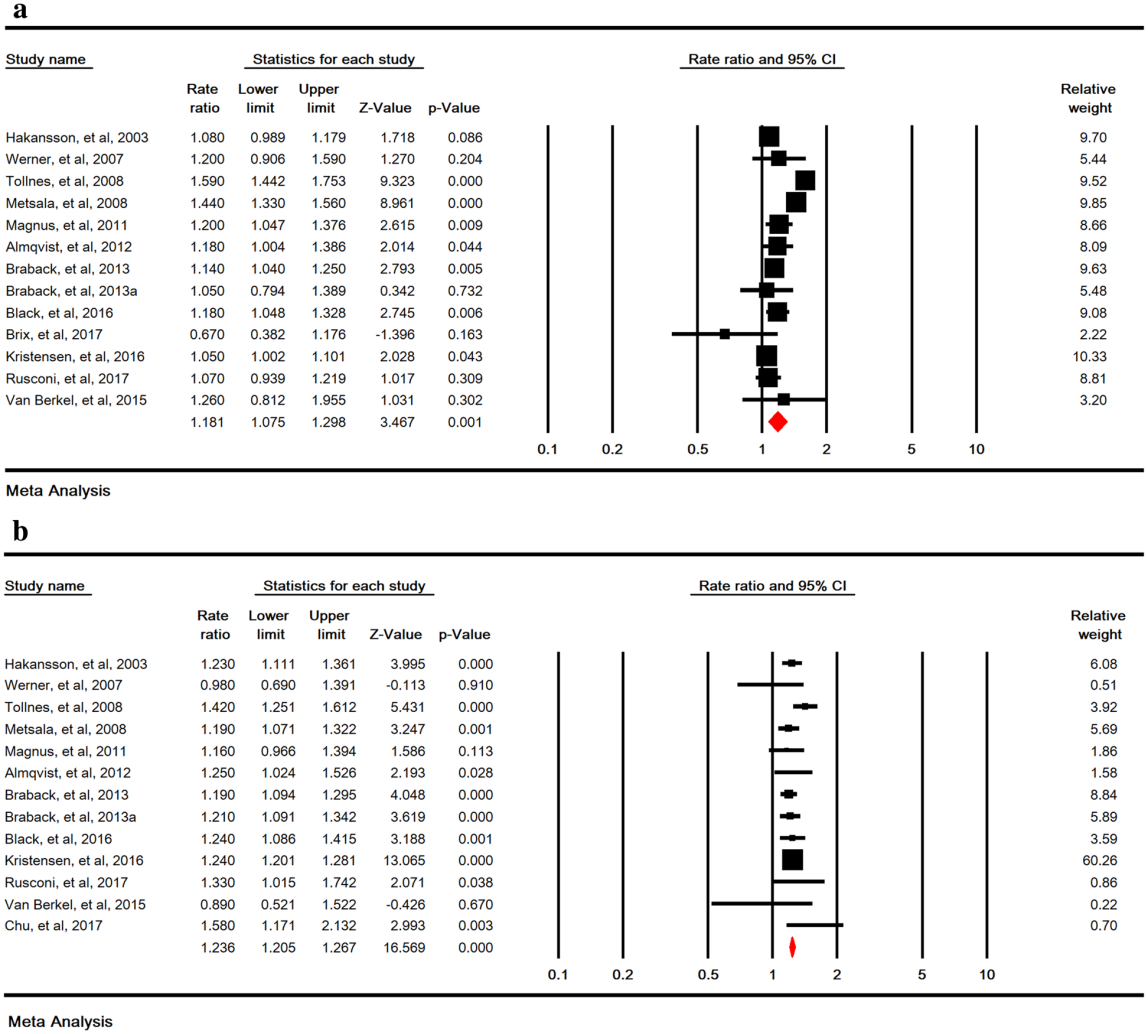
Relative risk of childhood asthma in emergency (**a**), and elective (**b**) cesarean section. A random-effects model was used for **a** and a fixed-effects model was used for **b**

**Fig. 5:**
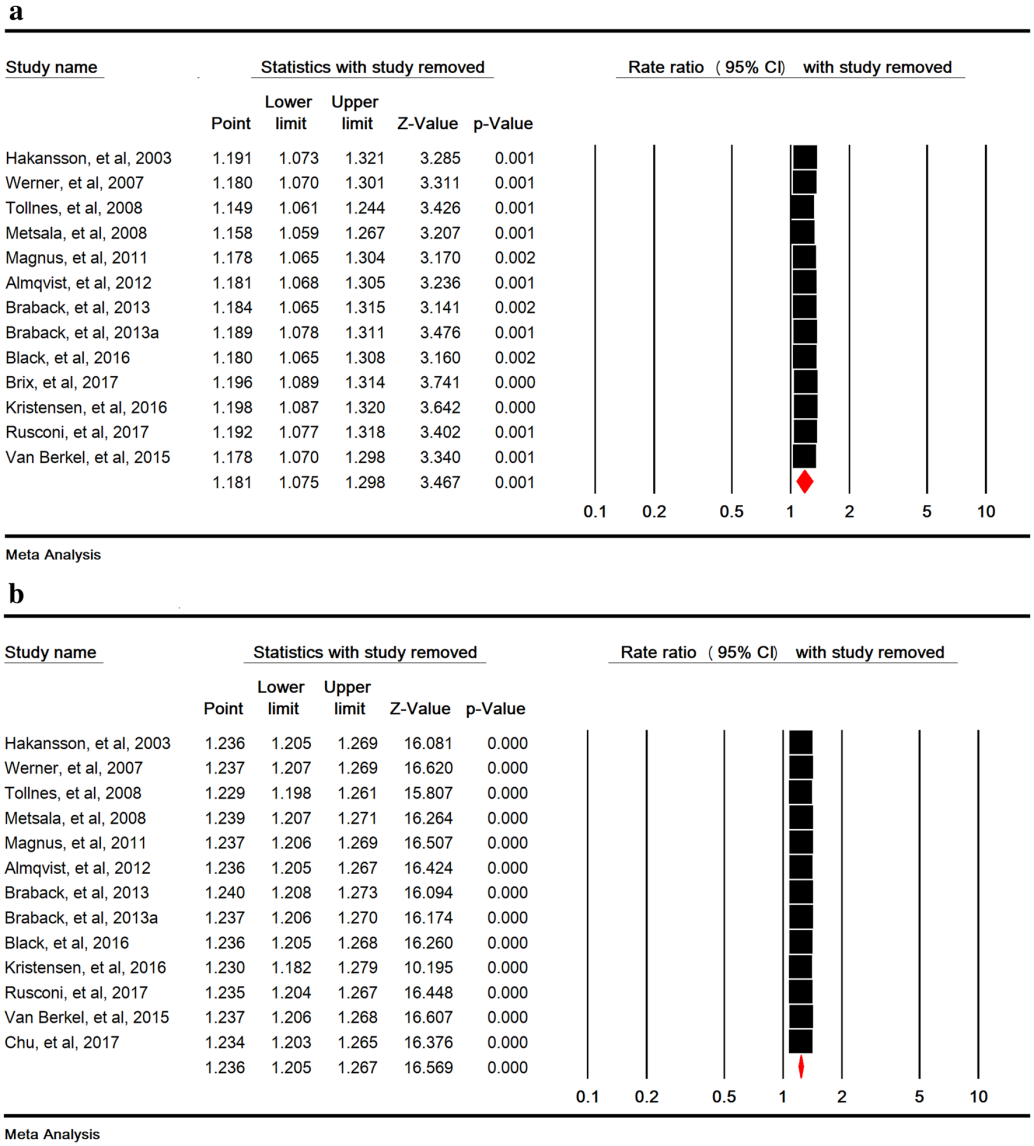
Sensitivity analysis for relative risks of childhood asthma in emergency (**a**), and elective (**b**) cesarean section

The association between emergency and elective SC and childhood asthma was significant with RR of 1.18 (95% CI 1.07–1.29, P < 0.001) in 13 studies and 1.23 (95% CI 1.20–1.26, P < 0.001) in 13 studies, respectively (Fig. 4a, b). Sensitivity analysis by removing one study at a time indicated that the overall estimate is robust (Fig. 5a, b).

### Publication bias

**Fig. 6:**
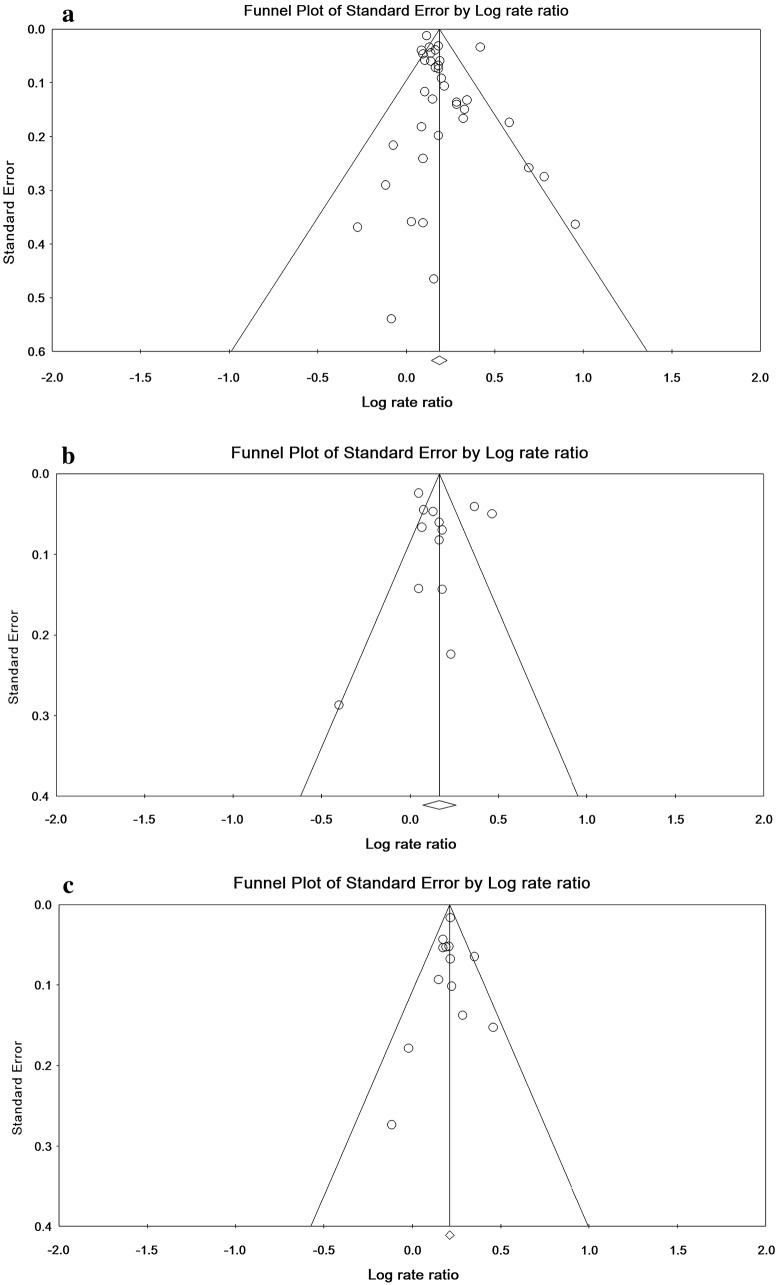
Funnel plot of relative risk of childhood asthma in overall (**a**), emergency (**b**) and elective (**c**) cesarean section

The statistical tests of publication bias were not significant for the RR of childhood asthma in the overall SC (Begg’s = 0.187, Egger = 0.569), emergency CS (Begg’s = 0.999, Egger = 0.291) and elective CS (Begg’s = 0.474, Egger = 0.607) (Fig. 6).

## Discussion

The present study is a systematic review and meta-analysis regarding CS and childhood asthma. In the final analysis of 37 studies, the relationship between CS and childhood asthma was statistically significant compared to vaginal delivery with a RR of 1.20 (95% CI 1.15–1.25). The results of different studies are reported to be contradictory; this relationship was significant in some studies [8–11, 21, 23, 24, 27, 28, 30, 32, 33, 35, 38, 40, 42–46] and it was not significant in others [12, 22, 25, 26, 29, 31, 34, 36, 37, 39, 41]. In a meta-analysis carried out in 2008, combination of 13 studies showed that cesarean delivery increases the risk of asthma [48]. According to the hygiene hypothesis, there are two possible causes: 1. Lack of contact of infants with mother’s bacteria during labor in CS, while these bacteria are necessary for the growth and development of the immune system. 2. Since infants are less in contact with stress hormone and chest pressure in CS, they suffer from more respiratory problems after birth. That’s because these mechanisms are associated with the emptying of the lungs from the amniotic fluid. This may have a negative effect on lung function in the long run [12, 13].

According to a study by Ghaffari et al. children with asthma have experienced shorter breastfeeding period, especially in the first 6 months of their lives, and this may be a potential mechanism to justify the role of delivery mode and breastfeeding duration on asthma incidence [49].

The present study also showed that there is a significant relationship between emergency and elective CS and childhood asthma, which is consistent with the studies of Tollånes et al. and Metsälä et al. [32, 33]. Some evidence suggest that perinatal metabolic changes may affect children’s immune system and increase the sensitivity of allergic diseases [50].

According to the findings of the present study, the association between CS and childhood asthma in developed countries is significant, which is consistent with the results of Thavagnanam’s meta-analysis [14].

Publication bias for studies on the relationship between CS and childhood asthma was evaluated according to Begg and Egger’s tests. The results showed that publication bias in the present study was not significant. It is assumed that the observed differences are due to different sampling and differences in the measured parameters in different societies.

## Weaknesses of the present study

(1) The omission of some studies, such as medical thesis and low-sample-size studies, due to their low quality; (2) the omission of several studies due to inharmonious reports and biased publications; (3) Europe was the context of most of the studies; (4) individual patients might have been included multiple times.

## Conclusion

This meta-analysis showed that CS (overall, elective, and emergency) increased the risk of childhood asthma.

This may reflect the hygiene hypothesis, according to which these children are likely to be less exposed to environmental microbes in early life. Future studies are needed to identify the effective factors in reducing this risk (especially in cases where elective or elective CS is not available) that can have important clinical and public health implications.

## Data Availability

Because the present article is a meta-analysis, the data extracted from the relevant articles from all over the world is available.

## Abbreviations

CS: caesarean section
UK: United Kingdom
SE: standard error
RR: relative risk
CI: confidence interval
